# Cohort Profile: The Nijmegen Exercise Study (NES)

**DOI:** 10.1093/ije/dyaf092

**Published:** 2025-06-25

**Authors:** Koen M van der Sluijs, Esmée A Bakker, Merle C A Schoofs, Janneke I A Vloet, André L M Verbeek, Maria T E Hopman, Dick H J Thijssen, Thijs M H Eijsvogels

**Affiliations:** Department of Medical BioSciences, Radboud University Medical Center, Nijmegen, The Netherlands; Department of Medical BioSciences, Radboud University Medical Center, Nijmegen, The Netherlands; Department of Primary and Community Care, Radboud University Medical Center, Nijmegen, The Netherlands; Department of Physical Education and Sports, Sport and Health University Research Institute (iMUDS), University of Granada, Granada, Spain; Department of Medical BioSciences, Radboud University Medical Center, Nijmegen, The Netherlands; Department of Medical BioSciences, Radboud University Medical Center, Nijmegen, The Netherlands; Department of IQ Health, Radboud University Medical Center, Nijmegen, The Netherlands; Department of Medical BioSciences, Radboud University Medical Center, Nijmegen, The Netherlands; Department of Medical BioSciences, Radboud University Medical Center, Nijmegen, The Netherlands; Research Institute for Sport and Exercise Sciences, Liverpool John Moores University, Liverpool, United Kingdom; Department of Medical BioSciences, Radboud University Medical Center, Nijmegen, The Netherlands

**Keywords:** exercise, physical activity, sedentary behaviour, cardiovascular disease, chronic disease, longitudinal, questionnaire, follow-up, lifestyle characteristics

Key Features

- The Nijmegen Exercise Study (NES) provides a longitudinal collection of data on lifestyle characteristics and the development and progression of chronic diseases. The NES is a population-based cohort enriched with highly physically active individuals. The cohort contains individuals both with and without chronic diseases, with physical activity levels ranging from inactive to extremely active.
- Baseline data collection is ongoing. Since 2011, baseline data have been collected in 23 643 participants with a mean age of 48.7 years (range: 18–94) of whom 46.0% are female. Annual follow-up data have been collected in 11 484 participants (48.6%) with a median follow-up duration of 8.0 (5.0, 10.9) years. Additional, centre-based evaluation is ongoing and has so far been performed in a subset of 1743 participants.
- Baseline and follow-up questionnaires provide trends in demographics, socioeconomic status, lifestyle characteristics, medical history, medication use, and historical and current physical activity and sedentary behaviour. Centre-based evaluation includes anthropometrics, blood pressure, arterial stiffness, carotid artery characteristics, venous blood biomarkers, accelerometer-based physical activity patterns, handgrip strength, and 4-metre gait speed.
- Data are available to scientific researchers upon request. Please contact the corresponding author (Thijs.Eijsvogels@radboudumc.nl) for data availability inquiries and visit our website for additional information (www.radboudumc.nl/nes).

## Why was the cohort set up?

The Nijmegen Exercise Study (NES) is a population-based cohort established in 2011 in Nijmegen, Netherlands. The objective of the NES is to study the relationship between lifestyle characteristics and the development and progression of chronic diseases. A physically active lifestyle reduces the risk of major non-communicable diseases, such as cardiovascular diseases, type 2 diabetes, certain types of cancer, and dementia [1–5]. Moreover, exercise is an effective treatment for many chronic diseases [6, 7].

Despite the abundant evidence for the health benefits of physical activity, certain knowledge gaps exist. Previous studies have often focused on associations between physical activity measured at a single time point and health outcomes at follow-up [1, 4, 8, 9]. This approach is prone to reverse-causation bias. Moreover, the intra-individual variability in physical activity levels over time introduces measurement error [10]. Obtaining repeated measurements, including objective measures of physical activity, may reduce the impact of these issues and enables the investigation of lifestyle changes across time. Another frequent limitation of population-based studies is the underrepresentation of participants with high physical activity volumes, as the majority of the Western population does not meet the World Health Organization (WHO) guidelines [2, 11, 12]. Accordingly, the certainty of findings concerning high(er) physical activity volumes is limited, leaving the dose–response relationship at high(er) volumes unclear.

The NES provides longitudinal data collection on lifestyle characteristics and the development and progression of chronic diseases in a population-based cohort enriched with highly physically active individuals. This cohort contains individuals both with and without chronic diseases, with physical activity levels ranging from inactive to extremely active. Annual questionnaires facilitate the longitudinal follow-up of lifestyle changes and reduce the risk of reverse-causation bias. Additional, centre-based evaluation was introduced in 2021 to objectify physical activity patterns and health parameters. Therefore, the NES provides opportunities to disentangle the relationship between lifestyle characteristics and the development and progression of chronic diseases.

## Who is in the cohort?

**Figure 1.**
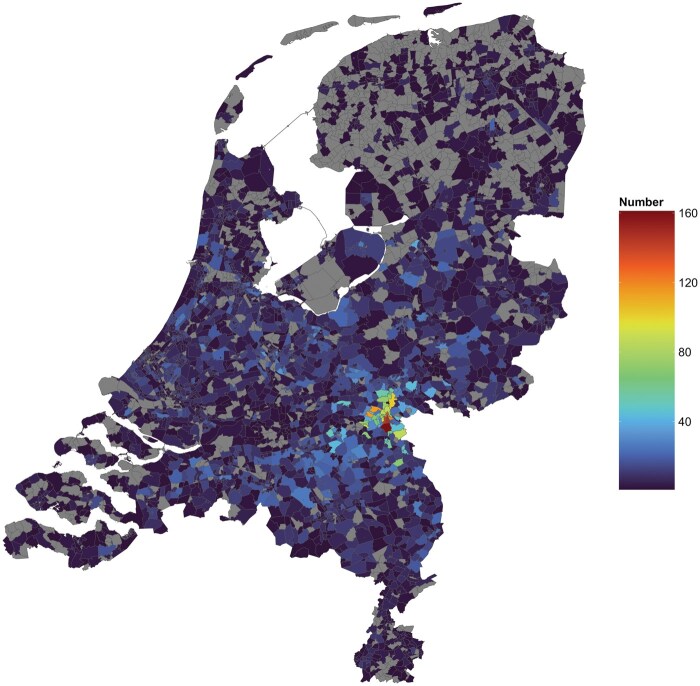
Number of NES participants per area of residence. A map of the Netherlands using a colour scale to depict the number of NES participants per postal code area.

The NES contains individuals participating in Dutch sports events (i.e. the International Nijmegen Four Days Marches and Seven Hills Run) as well as their family and friends. These sports events are organized by non-profit foundations (100+ and 40 editions, respectively), attracting ±40 000 Dutch and international participants annually. Study participants are recruited through newsletters amongst individuals partaking in these sports events. Individuals interested in participation can sign up via the NES website or e-mail. Baseline data collection started in 2011 and is ongoing; new participants are being enrolled annually. Inclusion criteria are an age of ≥18 years and Dutch residency and language proficiency. Individuals from across the Netherlands participate in the NES (Fig. 1). The study population is more physically active but otherwise comparable to the general Dutch adult population (Supplementary Table S1) [13]. Participants provide written informed consent prior to participation. Participants remain enrolled in the study until they opt out; participants can do so unconditionally. Baseline data have been collected in a total of 23 643 participants with a mean age of 48.7 (standard deviation: 13.5; range: 18–94) years, of whom 46.0% are female and of whom 79% adhere to the WHO guidelines on physical activity and sedentary behaviour [12] (Table 1 and Supplementary Table S2).

**Table 1.** Baseline participant characteristics of the NES.

Characteristic	All participants		Subset centre-based evaluation	
	*n *= 23 643	NA, %	*n *= 1743	NA, %
**Demographics**				
Age, years	48.7 (13.5)	0	54.8 (10.8)	0
Female sex	10 877 (46.0)	0	734 (42.1)	0
Ethnicity		1.5		1.2
White	22 844 (98.0)		1699 (98.7)	
Asian	216 (0.9)		8 (0.5)	
Black	56 (0.2)		4 (0.2)	
Other	184 (0.8)		11 (0.6)	
Marital status		0.4		0.3
Single	3632 (15.4)		177 (10.2)	
Married or civil partnership	15 019 (63.8)		1271 (73.1)	
Divorced	1036 (4.4)		75 (4.3)	
Living together	3456 (14.7)		180 (10.4)	
Widow or widower	403 (1.7)		35 (2.0)	
Education level		0.3		0
Primary education	239 (1.0)		13 (0.7)	
Lower vocational education	1655 (7.0)		101 (5.8)	
Secondary education	2175 (9.2)		178 (10.2)	
Secondary vocational education	4678 (19.8)		337 (19.3)	
Higher secondary education	2173 (9.2)		160 (9.2)	
Higher vocational education	7454 (31.6)		576 (33.0)	
University	4948 (21.0)		352 (20.2)	
Other	254 (1.1)		26 (1.5)	
Employment status		0.4		0.3
Employed	18 797 (79.8)		1309 (75.4)	
Not employed	4750 (20.2)		428 (24.6)	
Stay-at-home	57 (6.2)		6 (4.1)	
Student	74 (8.1)		3 (2.0)	
Volunteering	84 (9.2)		12 (8.2)	
Retired	600 (65.5)		106 (72.1)	
Unemployed	106 (11.6)		16 (10.9)	
Unfit for work	50 (5.5)		5 (3.4)	
Other	62 (6.8)		11 (7.5)	
Height, cm	175.9 (9.2)	0.2	175.5 (9.4)	0.2
Weight, kg	74.6 (13.0)	0.2	74.9 (13.8)	0.2
Waist circumference, cm	88.2 (11.6)	28.5	89.4 (11.4)	16.5
Hip circumference, cm^a^	97.7 (9.5)	83.9	97.7 (9.3)	74.4
Smoking behaviour		0.5		0.2
Current	1516 (6.4)		76 (4.4)	
Former	9122 (38.8)		739 (42.5)	
Never	12 880 (54.8)		925 (53.2)	
Alcohol consumption, glasses/week	4.0 [2.0, 8.0]	13.3	5.0 [2.0, 10.0]	11.3
**Medical history**				
Cancer	1132 (5.2)	7.2	100 (6.2)	7.2
Bladder	25 (0.1)		0 (0.0)	
Bone	5 (0.0)		0 (0.0)	
Breast	129 (0.6)		10 (0.6)	
Cervical	37 (0.2)		1 (0.1)	
Colorectal	47 (0.2)		6 (0.4)	
Lung	12 (0.1)		0 (0.0)	
Lymphoma	46 (0.2)		0 (0.0)	
Pancreatic	3 (0.0)		0 (0.0)	
Prostate	56 (0.3)		5 (0.3)	
Skin	250 (1.1)		29 (1.8)	
Testicular	16 (0.1)		1 (0.1)	
Thyroid	13 (0.1)		0 (0.0)	
Cardiovascular diseases				
Myocardial infarction	359 (1.7)	8.4	40 (2.5)	7.9
Heart failure^a^	219 (1.8)	47.2	21 (2.3)	48.0
Stroke	281 (1.3)	8.8	30 (1.9)	8.7
Thrombosis^a^	74 (1.4)	77.7	9 (1.7)	70.3
Atrial fibrillation^a^	103 (1.9)	77.5	14 (2.7)	70.0
Hypertension	3292 (15.0)	7.0	300 (18.4)	6.5
Hypercholesterolaemia	2494 (11.4)	7.4	255 (15.7)	6.8
Diabetes mellitus	612 (2.8)	8.6	45 (2.8)	8.5
Resuscitation^a^	23 (0.4)	77.2	4 (0.8)	69.7
Asthma, chronic bronchitis, or COPD	1935 (8.9)	8.3	120 (7.5)	8.0
Kidney disease^a^	43 (1.3)	85.7	9 (2.0)	74.1
Neurological diseases				
Dementia or Alzheimer’s disease^a^	0 (0.0)	97.8	0 (0.0)	91.7
Epilepsy^a^	123 (1.0)	47.3	6 (0.7)	48.7
Parkinson’s disease^a^	13 (0.1)	47.5	1 (0.1)	48.7
Depression	1720 (8.0)	8.5	126 (7.9)	8.3
Rheumatic disease	600 (2.8)	8.7	54 (3.4)	8.4
Arthrosis^a^	792 (6.3)	46.8	91 (10.0)	47.8
Osteoporosis	456 (2.1)	8.8	38 (2.4)	8.7
Allergy^a^	2638 (20.9)	46.6	187 (20.7)	48.2
Immunological disease^a^	140 (1.1)	47.3	14 (1.6)	48.5
Thyroid disease^a^	462 (3.7)	47.0	32 (3.5)	48.3
**Physical activity and sedentary behaviour**				
Habitual physical activity, MET-minutes/week	1746 [747, 3324]	0.2	1980 [840, 3607]	0.1
Habitual resistance exercise^a^	634 (28.3)	90.5	109 (31.7)	80.3
Habitual sedentary behaviour, hours/day^a^	9.1 (3.6)	85.4	9.2 (3.5)	73.4

Variables are reported as mean (standard deviation), median [Q_25_, Q_75_], or number (percentage).

COPD, chronic obstructive pulmonary disease; MET, metabolic equivalent of task; NA, not available.

a Variable introduced at a later stage.

Since 2021, a subset of 1743 NES participants have visited the research centre [Radboud university medical center(Radboudumc), Nijmegen, The Netherlands] to undergo additional, centre-based evaluation (Table 2 and Supplementary Table S2). Approximately 400 participants are invited for centre-based evaluation annually; pregnancy is the only exclusion criterion. Currently (2025), we have oversampled participants with cardiovascular risk factors and/or disease to investigate associations of lifestyle characteristics with cardiovascular health (Table 1).

**Table 2.** Participant characteristics assessed during centre-based evaluation.

	Subset centre-based evaluation	
	*n *= 1743	NA, %
Anthropometrics		
Body mass index, kg/m^2^	24.7 (3.5)	0.1
Body fat mass, kg	19.5 (7.8)	3.1
Fat free mass, kg	55.6 (10.5)	3.1
Skeletal muscle mass, kg	28.5 (7.0)	3.1
Percentage body fat, %	25.7 (7.8)	3.1
Cardiovascular risk factors		
Systolic blood pressure, mmHg	137.7 (17.6)	0.5
Diastolic blood pressure, mmHg	83.2 (9.6)	0.5
Resting heart rate, beats/minute	60.3 (10.3)	0.5
Carotid–femoral pulse wave velocity, m/s^a^	8.6 (3.1)	56.5
Carotid stiffness index, a.u.^a^	6.3 (3.0)	56.8
Carotid pressure–strain elastic modulus, kPa^a^	86.2 (43.4)	56.8
Carotid artery reactivity, %^a^	2.4 [1.4, 3.5]	84.5
Venous blood biomarkers		
Total cholesterol, mmol/L	5.2 [4.5, 5.9]	1.5
High-density lipoprotein, mmol/L	1.6 [1.3, 1.9]	1.5
Low-density lipoprotein, mmol/L	3.0 [2.4, 3.6]	1.5
Triglycerides, mmol/L	1.0 [0.8, 1.3]	1.5
Glucose, mmol/L	4.9 [4.6, 5.2]	10.6
Insulin, mIU/ml	3.5 [2.0, 6.2]	1.3
Creatinine, mmol/L	77 [67, 86]	1.5
High-sensitive cardiac troponin I, ng/L	4.2 [2.6, 7.5]	1.7
Amino-terminal pro-B-type natriuretic peptide, pmol/L	10.0 [6.0, 18.0]	1.4
C-reactive protein, mg/L	4.0 [4.0, 4.0]	1.5
Physical function		
Accelerometry		
Light-intensity physical activity, minutes/day	279 (79)	2.9
Moderate-to-vigorous physical activity, minutes/day	106 (40)	2.9
Standing time, hours/day	3.9 (1.2)	2.9
Sitting time, hours/day	9.2 (1.5)	2.9
Sleeping time, hours/day	8.4 (1.3)	2.9
Step count, steps/day	13 643 (4977)	2.9
Handgrip strength, kg	38 (12)	0.1
Four-metre gait speed, km/hour	5.6 (0.8)	0.3

Variables are reported as mean (standard deviation), median [Q_25_, Q_75_], or number (percentage).

NA, not available.

a Variable collected in a subset of participants.

## How often have they been followed up?

NES participants receive annual follow-up questionnaires. Participants are encouraged to complete the annual questionnaires, but failing to do so does not result in exclusion from further participation. Currently (2025), follow-up data have been collected in 11 484 (48.6%) participants. The median (Q_25_, Q_75_) follow-up duration is 8.0 (5.0, 10.9) years and follow-up data of >5 years are available in 8517 (36.0%) participants. Follow-up objective measurements have not yet started, as these are planned every 5 years.

## What has been measured?

**Figure 2.**
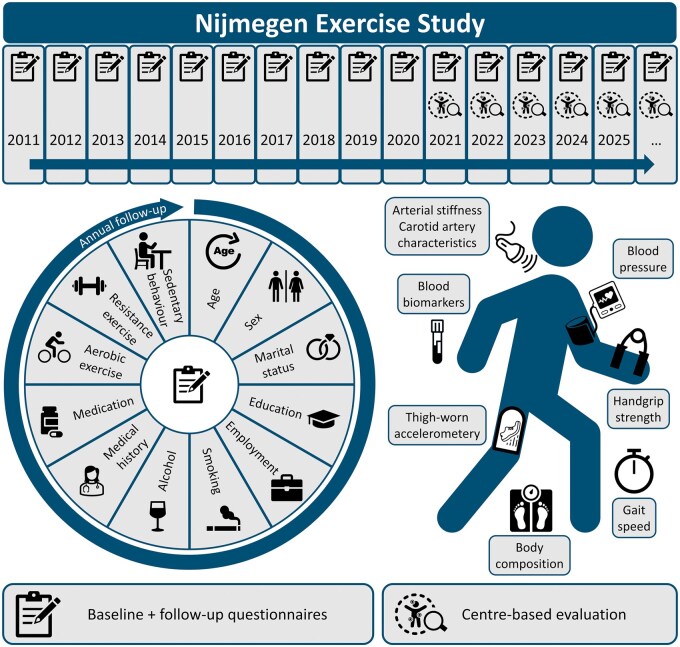
Overview of the NES. A graphical overview showing how and when variables are collected in the NES.

The NES consists of baseline and annual follow-up questionnaires sent by e-mail and, in a subset of participants, centre-based evaluation performed at Radboudumc (Fig. 2 and Supplementary Table S3). Additionally, participants can provide access to personal data from third parties, such as medical files, health insurance claim data, and data of national registers (e.g. cause of death via Statistics Netherlands).

### Baseline and follow-up questionnaires

Demographics include date of birth, sex, ethnicity, marital status, education level, and employment status. Participants fill out their height, weight, waist and hip circumference, smoking behaviour, and alcohol consumption. Pregnancy-related data are collected in female participants. History of 22 medical conditions is assessed, including the age at the time of the event/diagnosis. The type and dosage of medication used are collected via open-text fields, including the time of use for cholesterol-lowering medication. The volume and intensity of habitual physical activity are assessed by using the Short QUestionnaire to ASsess Health-enhancing physical activity (SQUASH) [14]. Since 2022, the type, volume, and intensity of habitual resistance exercise have been assessed by using the Muscle-Strengthening Exercise Questionnaire (MSEQ) [15]. Between 2016 and 2021, a shortened, adapted version of the MSEQ has been used. Habitual sedentary behaviour is quantified by using the Sedentary Behavior Questionnaire (SBQ) [16]. The baseline questionnaire also assesses historical physical activity and sedentary behaviour patterns. For each age range (i.e. 17–29, 30–49, 50–64, and >65 years old) reached or passed at the time of completing the baseline questionnaire, participants fill out the average time and intensity spent on exercise and sedentary behaviour whilst being in that age range. Moreover, in 2018 and 2022, a subset (*n *= 3270) completed the Cognitive Online Self‐Test Amsterdam (COST‐A)—an online, self-administered test of cognitive functioning [17].

### Centre-based evaluation

At Radboudumc, the participants’ physical activity and health status are objectively assessed. Measurements focus on cardiovascular risk and physical function, given their association with lifestyle characteristics. Prior to their visit, participants are instructed to fast for ≥4 hours, refrain from strenuous exercise for 24 hours, and refrain from alcohol and caffeine for 18 hours to ensure valid assessment of vascular function and arterial stiffness [18–20].

#### Anthropometrics

Height and body mass are measured (222 and 813, Seca, Hamburg, Germany) and body mass index (BMI) is calculated. Body composition is assessed by using bioelectrical impedance analysis and quantified by using body fat mass, fat free mass, skeletal muscle mass, and percentage body fat. Single-frequency analysis (1500, Bodystat, Douglas, Isle of Man) was used in 2021 (*n *= 528); multi-frequency analysis (770, InBody, Seoul, South Korea) has been used since then (*n *= 820).

#### Cardiovascular risk factors

##### Blood pressure and heart rate

Non-invasive brachial blood pressure and heart rate are measured by using an automatic sphygmomanometer (M3, OMRON, Kyoto, Japan) after 10 minutes of supine rest. Measurements are taken twice on the left arm and once on the right arm. In case of discrepancy between measurements (difference of >10 mmHg systolic or >5 mmHg diastolic blood pressure), a fourth measurement is taken on the right arm.

##### Central and local arterial stiffness

Central and local carotid arterial stiffness are assessed by using A-mode ultrasound (ARTSENS Plus, Healthcare Technology Innovation Center, Indian Institute of Technology Madras, Chennai, India) [21, 22]. Measurements are performed after 10 minutes of supine rest. Non-invasive left brachial blood pressure and heart rate are obtained via the integrated sphygmomanometer. Local arterial stiffness of the left common carotid artery is derived from the arterial distensibility and blood pressure, and expressed by using stiffness index Beta and pressure–strain elasticity *E*_P_. Central arterial stiffness expressed as the carotid–femoral pulse wave velocity (cfPWV) is estimated by using the pulse transit time and the effective path length is measured according to established methods [19, 20]. The pulse transit time is derived from simultaneous recordings of ultrasound-based carotid artery distensibility and cuff-based femoral artery blood pressure, averaged over 10 cardiac cycles. Arterial stiffness parameters are computed automatically by the device.

##### Carotid artery characteristics

Carotid artery characteristics, including arterial wall thickness, intima-media thickness, and carotid artery reactivity, were assessed by using carotid ultrasound in a subset (*n *= 271) in 2021. Carotid artery reactivity assesses the response of the carotid artery diameter to sympathetic stimulation by using a cold pressor test [23]. The ultrasound recording is processed afterwards (BloodFlow Software, version 4.0, National Instruments LabVIEW, Austin, TX, USA). A description of the measurement procedure, processing steps, and outcome measures is provided elsewhere [23].

#### Venous blood biomarkers

Venous blood is drawn (SST II Advance and PST II Advance, BD, Franklin Lakes, NJ, USA), coagulated for 45–60 minutes (SST II Advance only), and centrifuged at 3000 revolutions/minute for 10 minutes at 4°C. Serum and plasma are transferred to 2-ml microtubes and stored at –80°C. A part of the serum is used to analyse the following biomarkers: total, high-density lipoprotein and low-density lipoprotein cholesterol, triglycerides, glucose hexokinase, insulin, creatinine, high-sensitive cardiac troponin I, amino-terminal pro-B-type natriuretic peptide, and C-reactive protein. Analyses are performed batchwise on Atellica (IMMULITE 2000 for insulin) analysers (Siemens Healthcare, Erlangen, Germany). The remaining samples are stored at Radboudumc Biobank and available for future use.

#### Physical function

##### Accelerometer-based physical activity

Ambulant physical activity and sedentary behaviour are assessed objectively by using thigh-worn triaxial accelerometry (activPAL3 micro, PAL Technologies, Glasgow, UK). Measurements are conducted 24 hours/day over an 8-day period with a sampling rate of 20 Hz. Participants file a sleep/wake/activity log during this period to facilitate automated analysis. PALconnect software (version 8, PAL Technologies, Glasgow, UK) extracts and saves the data in a proprietary file format. PALbatch software reads the proprietary file; classifies the recording into epochs of sedentary, standing, or stepping behaviour; and saves the classifications in a comma-separated values file. Additionally, raw triaxial acceleration data are extracted from the proprietary file.

The classifications file is analysed by using a modified version of the script by Winkler *et al.* [24, 25] via the Statistical Analysis System (version 9.4, SAS Institute, Cary, NC, USA). Current output variables include but are not limited to time spent in light-intensity physical activity and moderate-to-vigorous physical activity, standing, sitting, sleeping, and step count. Furthermore, daily amounts of physical activity and sedentary bouts of various lengths are computed.

##### Handgrip strength

Peak handgrip strength of the non-dominant hand is measured three times separated by 1-minute intervals while seated by using a Jamar hydraulic hand dynamometer following the method by Webb *et al.* [26]. The highest value is used for analysis.

##### Four-metre gait speed

The preferred gait speed is measured twice over a 4-metre stretch with 2-metre acceleration and deceleration zones on either side to ensure a stable speed. The fastest attempt is used for analysis, following in part the Short Physical Performance Battery protocol [27].

## What has it found?

Articles using data of the NES have been published in peer-reviewed journals. We reported that sedentary behaviour is prevalent in physically active individuals [28] and the total sedentary time may be positively associated with cognitive functioning [29]. Moreover, correlates for high sedentary behaviour volumes may vary across domains of sitting, such as transportation, occupation, and leisure time. During the coronavirus disease 2019 (COVID-19) pandemic, data of follow-up questionnaires revealed reductions in physical activity levels at the time of restrictive policy measures compared with pre-pandemic questionnaires [30]. Using data of the centre-based evaluations, we compared participants 6 months after a COVID-19 episode to participants who were free of COVID-19. We found no differences in accelerometer-based physical activity levels or cardiovascular risk factors, yet one in three participants experienced residual COVID-19 complaints [31].

Other studies have focused on a better understanding of cardiovascular risk. We demonstrated that new anthropometrics (i.e. body shape index and body roundness index) are not superior to BMI or waist circumference in identifying cardiovascular health status [32]. Furthermore, we retrospectively investigated exercise dose over a median of 32 years and found a curvilinear association between exercise patterns and cardiovascular morbidity [33]. These findings suggested that even low exercise volumes are associated with a lower prevalence of cardiovascular diseases. Moreover, we demonstrated no association between statin use and prevalence of exercise-related injuries in amateur runners [34], suggesting that statin users can continue normal physical activity without concern for an elevated risk of injuries. Using data of the centre-based evaluations, we compared arterial stiffness between NES participants and matched volunteers from India and found ethnicity-related differences in arterial stiffness [35], suggesting that arterial stiffness may contribute to the difference in cardiovascular risk between ethnicities. Moreover, we found an association between objectively measured sitting time and local carotid but not central arterial stiffness [36].

Finally, the centre-based data collection is shared within ProPASS [37]—an international collaboration of cohorts using thigh-worn accelerometry. Studies using pooled ProPASS data suggested that time reallocation towards moderate-to-vigorous physical activity is beneficial for cardiometabolic health [38]; another study quantified the dose–response relationships between physical activity type and posture and cardiometabolic health markers [39].

## What are the main strengths and weaknesses?

A main strength is that the NES population covers the full physical activity spectrum, from inactive to extremely active [34]. This provides the means to study the entire dose–response relationship between physical activity and health outcomes. Another strength is that the NES provides an elaborate, longitudinal collection of physical activity data, including data on aerobic and resistance exercise, domain-specific (e.g. leisure-time and work-related) physical activity and sedentary behaviour [28, 29], and historical physical activity and sedentary behaviour [33]. Objective accelerometry expands the data collection [31, 37–39]. The NES collects data on a wide range of health parameters and is not confined to a single disease outcome. Across thousands of participants, the prevalence of >20 diagnoses is monitored annually along with medication use and healthcare consumption. This creates opportunities for studying the relationship between physical activity and the development and progression of diseases.

A weakness of the NES is the sampling. New participants sign up themselves, so digitally skilled individuals who value scientific research may be oversampled. The subset undergoing centre-based evaluation may over-represent participants living near Nijmegen for practical reasons. Moreover, causality cannot be inferred, because of the observational design. Furthermore, objective measurements were introduced in 2021 when 10 years of questionnaire data had already been collected. The subset that has undergone centre-based evaluation is therefore limited but is expected to expand by 400 each year. Finally, the NES is a monocentre study and is restricted to the inclusion of Dutch individuals because of the questionnaire language and linkage to Dutch registers. However, individuals from across the Netherlands participate in the NES.

## Can I get hold of the data? Where can I find out more?

Data of the NES are internationally available to scientific researchers upon request. Researchers can submit a research proposal to the corresponding author by e-mail, detailing which data are requested and for which purpose. Proposals will be reviewed on scientific quality and methodology by the NES principal investigators (E.A.B., M.T.E.H., D.H.J.T., T.M.H.E.) and collaboration terms and conditions will be discussed. Upon approval, NES data and linked data will be made accessible via a secure, virtual workspace that includes standard analysis software. Currently, no access fees apply, but an operating fee will be charged to cover the virtual workspace costs (generally <€ 100/month). Contact the corresponding author (Thijs.Eijsvogels@radboudumc.nl) for data availability inquiries and visit our website for more information (www.radboudumc.nl/nes).

## Ethics approval

This study (NL36743.091.11) was approved by the local Committee on Research Involving Human Subjects of the region Arnhem and Nijmegen, Netherlands, and the study was conducted in accordance with the Declaration of Helsinki. All participants provided written informed consent.

## Supplementary Material

dyaf092_Supplementary_Data

## Data Availability

See ‘Can I get hold of the data? Where can I find out more?’ above.
