# Supplementary material for: Cohort Profile: The Nijmegen Exercise Study (NES)
Source: Int J Epidemiol. 2025 Jun 25;54(4):dyaf092. doi: 10.1093/ije/dyaf092 (PMC12198492; doi:10.1093/ije/dyaf092)
Supplement: dyaf092_Supplementary_Data [file dyaf092_supplementary_data.docx]

**Supplementary Tables**

**Supplementary Table S1.** Characteristics of the Nijmegen Exercise Study population and the general Dutch adult population.

| **Characteristic** | **Nijmegen Exercise Study** | | **General Dutch adult population** |
| --- | --- | --- | --- |
|  | *n*=23 643 | NA, % | *n*=17 811 291^1,ˆ^ |
| Age, years | 48.7 (13.5) | 0 | 48.4 (N/A)^1,†^ |
| Female sex | 10 877 (46.0) | 0 | 6 752 855 (50.6)^1,†^ |
| Adherence to WHO guidelines* | 18 639 (79) | 0.2 | N/A (50)^2,‡^ |
| Current smoker | 1516 (6.4) | 0.5 | N/A (19)^2,‡^ |
| Cancer | 1132 (5.2) | 7.2 | 925 431 (5.2)^3,ˆ^ |
| Myocardial infarction | 359 (1.7) | 8.4 | 264 000 (1.5)^4,ˆ^ |
| Heart failure^~^ | 219 (1.8) | 47.2 | 246 500 (1.4)^4,ˆ^ |
| Stroke | 281 (1.3) | 8.8 | 543 200 (3.0)^4,ˆ^ |
| Hypertension | 3292 (15.0) | 7.0 | 2 809 500 (15.7)^4,ˆ^ |
| Hypercholesterolaemia | 2494 (11.4) | 7.4 | 1 574 500 (8.8)^4,ˆ^ |
| Diabetes mellitus | 612 (2.8) | 8.6 | 1 178 200 (6.6)^4,ˆ^ |
| Depression | 1720 (8.0) | 8.5 | 515 600 (2.9)^4,ˆ^ |
| Rheumatic disease | 600 (2.8) | 8.7 | 277 100 (1.6)^4,ˆ^ |

Variables are reported as mean (standard deviation) or number (percentage). N/A: not available, WHO: World Health Organization.
^*^ World Health Organization guidelines on physical activity and sedentary behaviour^5^; adherence defined as habitual physical activity volumes greater than 600 metabolic equivalent of task-minutes per week.
^˜^ Variable introduced at a later stage.
^†^ Mean age and number of females of the general Dutch adult population estimated over the period 2011-2024, the standard deviation of the age of general Dutch adult population is not available.
^‡^ Absolute number of people in the general Dutch adult population not available for this variable. ^ˆ^ Numbers as of 1 January 2023.

**References**

1. Centraal Bureau voor de Statistiek. *Bevolking op 1 januari en gemiddeld; geslacht, leeftijd en regio*. 16 October 2024 [cited; Available from: <https://opendata.cbs.nl/#/CBS/nl/dataset/03759ned/table?dl=B4E29>

2. Centraal Bureau voor de Statistiek, Rijksinstituut voor Volksgezondheid en Milieu. *Gezondheidsenquête/Leefstijlmonitor*. 2023 [cited 2024 September 30]; Available from: <https://www.rivm.nl/leefstijlmonitor>

3. Integraal Kankercentrum Nederland. *Nederlandse Kankerregistratie*. 2023 [cited 2025 29 January]; Available from: <https://nkr-cijfers.iknl.nl/viewer/prevalentie-per-jaar?language=nl_NL&viewerId=3d4692aa-a8ed-4193-a56a-55a75aa1fdfb>

4. Nederlands instituut voor onderzoek van de gezondheidszorg. *Nivel Zorgregistraties eerste lijn*. 2023 [cited 2025 29 January]; Available from: <https://www.vzinfo.nl/>

5. World Health Organization. WHO guidelines on physical activity and sedentary behaviour. Geneva: World Health Organization; 2020.

**Supplementary Table S2.** Overview of inclusions in the Nijmegen Exercise Study per year.

| **Year^*^** | ***n* enrolled** | ***n* included in centre-based evaluation** |
| --- | --- | --- |
| 2011 | 9928 | Not applicable |
| 2012 | 4388 | Not applicable |
| 2013 | 3732 | Not applicable |
| 2014 | 1989 | Not applicable |
| 2015 | 1357 | Not applicable |
| 2016 | 1095 | Not applicable |
| 2017 | 604 | Not applicable |
| 2018 | 0 | Not applicable |
| 2019 | 201 | Not applicable |
| 2020 | 165 | Not applicable |
| 2021 | 56 | 572 |
| 2022 | 26 | 472 |
| 2023 | 102 | 373 |
| 2024 | 0 | 359 |
| Total | 23 643 | 1776 |

^*^ Participants were included in July and November.

**Supplementary Table S3.** Data catalogue of the Nijmegen Exercise Study.

| **Category** | **Subcategory** | **Variables** |
| --- | --- | --- |
| Baseline and follow-up questionnaires | Demographics and lifestyle characteristics | Age [years]; sex [m, f]; ethnicity [White, Asian, Black, other]; marital status [single, married or civil partnership, divorced, living together, widow or widower]; education level [primary education, lower vocational education, secondary education, secondary vocational education, higher secondary education, higher vocational education, university, other]; employment status [employed, stay-at-home, student, volunteering, retired, unemployed, unfit for work, other]; height; weight; waist circumference; hip circumference; smoking behaviour [current user, former user, never used]; alcohol consumption [glasses/week]; pregnancy [history of pregnancy, number of pregnancies, pregnancy-related conditions]. |
|  | Medical history and medication use | Answering options for the following conditions [yes, no; if yes: age at time of event or diagnosis]: cancer [if yes: type of cancer], myocardial infarction, heart failure, stroke, thrombosis, atrial fibrillation, hypertension, hypercholesterolaemia, diabetes mellitus, resuscitation, asthma, chronic bronchitis, chronic obstructive pulmonary disease, kidney disease, dementia, Alzheimer’s disease, epilepsy, Parkinson’s disease, depression, rheumatic disease, arthrosis, osteoporosis, allergy [if yes: type of allergy], immunological disease, thyroid disease; type of medication; dosage of medication. |
|  | Habitual physical activity | Walking/bicycling to work/school [volume; intensity]; light-to-moderate-intensity activity at work/school [volume]; vigorous-intensity activity at work/school [volume]; light-to-moderate-intensity household activity [volume]; vigorous-intensity household activity [volume]; leisure time walking [volume; intensity]; leisure time bicycling [volume; intensity]; gardening [volume; intensity]; odd jobs [volume; intensity]; for each sport separately: [volume; intensity]; number of days/week with more than 30 minutes spent being physically active. |
|  | Habitual resistance exercise | Resistance exercise [yes, no]; type of resistance exercise [bodyweight exercises, free weights, weight machines, other]; volume [number of training sessions/week, hours/training session]; intensity, questions repeated for upper body and lower body: [light weights and few (<12) repetitions, light weights and many (≥12) repetitions, heavy weights and few(<12) repetitions, heavy weights and many (≥12) repetitions]. |
|  | Habitual sedentary behaviour | Time spent sitting [hours/day] on the following activities on a typical weekday and on a typical weekend day: eating or drinking; watching television; working; using electronic devices; transport; listening to music; telephoning; reading; playing an instrument or doing artwork. |
|  | Historical physical activity and sedentary behaviour patterns | Questions repeated for each age range (17-29, 30-49, 50-64, >65 years old): exercise [yes, no]; aerobic exercise [volume; intensity]; resistance exercise [volume]; time spent sitting [hours/day] in the following domains on a typical weekday and on a typical weekend day: at work; at home; during transport. |
|  | Cognitive functioning | Z-scores on the following tasks: orientation; digit-sequence learning; immediate word recall; connecting numbered dots; letter-number alteration; free delayed word recall; delayed word recognition; word pairs immediate recall; word pairs recognition; semantic comprehension. |
| Centre-based evaluation | Anthropometrics | Height [m]; body mass [kg]; body mass index [kg/m^2^]; body fat mass [kg]; fat free mass [kg]; skeletal muscle mass [kg]; percentage body fat [%]. |
|  | Cardiovascular risk factors | Non-invasive brachial systolic and diastolic blood pressure [mmHg]; resting heart rate [beats/minute]; central arterial stiffness (carotid-femoral pulse wave velocity [m/s]); local carotid arterial stiffness (stiffness index Beta [a.u.] and pressure-strain elastic modulus E_P_ [kPa]); carotid arterial wall thickness [mm]; carotid intima-media thickness [mm]; carotid artery reactivity (baseline diameter [cm]; area under the curve [cm*s]; peak diameter [cm]; time to peak [s]; maximum reactivity [cm]; maximum reactivity [%]; slope of the curve [mm/s]; duration of response [s]). |
|  | Venous blood biomarkers | Full cholesterol profile (total cholesterol; high-density lipoprotein; low-density lipoprotein; triglycerides [mmol/L]); glucose hexokinase [mmol/L]; insulin [mIU/mL]; creatinine [mmol/L]; high-sensitive cardiac troponin I [ng/L]; amino-terminal pro-B-type natriuretic peptide [pmol/L]; C-reactive protein [mg/L]; serum and plasma samples stored at Radboudumc Biobank for future use. |
|  | Physical function | Accelerometery-based physical activity patterns, including: light-intensity physical activity [minutes/day]; moderate-to-vigorous physical activity [minutes/day]; standing time [hours/day]; sitting time [hours/day]; sleeping time [hours/day]; step count [steps/day]; number of physical activity bouts; number of sedentary bouts; handgrip strength [kg]; four-metre gait speed [km/hour]. |
